# *In Vivo* Diffusion Tensor Imaging in Acute and Subacute Phases of Mild Traumatic Brain Injury in Rats

**DOI:** 10.1523/ENEURO.0476-19.2020

**Published:** 2020-06-15

**Authors:** Isabel San Martín Molina, Raimo A. Salo, Ali Abdollahzadeh, Jussi Tohka, Olli Gröhn, Alejandra Sierra

**Affiliations:** A.I. Virtanen Institute for Molecular Sciences, University of Eastern Finland, Kuopio FI-70211, Finland

**Keywords:** axonal damage, cell counting, diffusion tensor imaging, inflammation, mild traumatic brain injury, secondary damage, structure tensor

## Abstract

Mild traumatic brain injury (mTBI) is the most common form of TBI with 10–25% of the patients experiencing long-lasting symptoms. The potential of diffusion tensor imaging (DTI) for evaluating microstructural damage after TBI is widely recognized, but the interpretation of DTI changes and their relationship with the underlying tissue damage is unclear. We studied how both axonal damage and gliosis contribute to DTI alterations after mTBI. We induced mTBI using the lateral fluid percussion (LFP) injury model in adult male Sprague Dawley rats and scanned them at 3 and 28 d post-mTBI. To characterize the DTI findings in the tissue, we assessed the histology by performing structure tensor (ST)-based analysis and cell counting on myelin-stained and Nissl-stained sections, respectively. In particular, we studied the contribution of two tissue components, myelinated axons and cellularity, to the DTI changes. Fractional anisotropy (FA), mean diffusivity (MD), and axial diffusivity (AD) were decreased in both white and gray matter areas in the acute phase post-mTBI, mainly at the primary lesion site. In the subacute phase, FA and AD were decreased in the white matter, external capsule, corpus callosum, and internal capsule. Our quantitative histologic assessment revealed axonal damage and gliosis throughout the brain in both white and gray matter, consistent with the FA and AD changes. Our findings suggest that the usefulness of *in vivo* DTI is limited in its detection of secondary damage distal to the primary lesion, while at the lesion site, DTI detected progressive microstructural damage in the white and gray matter after mTBI.

## Visual Abstract

**Figure:**
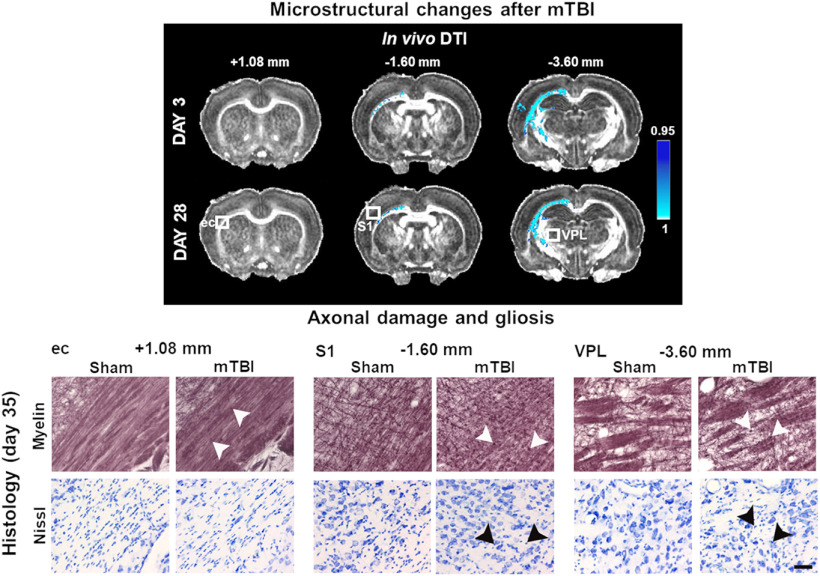

## Significance Statement

Mild traumatic brain injury (mTBI) is a major health problem worldwide with an unclear diagnosis. Using the lateral fluid percussion (LFP) injury model in rats, we induced mTBI to assess the potential of *in vivo* diffusion tensor imaging (DTI) for non-invasively detecting progressive microstructural tissue damage. To interpret the changes observed in DTI, we performed extensive quantitative histologic assessment of the tissue microstructure. From the acute to subacute phases after mTBI, *in vivo* DTI detected progressive microstructural tissue alterations in the white and gray matter associated with axonal damage and gliosis. Although *in vivo* DTI failed to detect secondary tissue damage far from the primary lesion, these findings provide new insights for detecting mild tissue damage using *in vivo* DTI.

## Introduction

Mild traumatic brain injury (mTBI) is the most common form of TBI, affecting 42 million people worldwide per year (Gardner and Yaffe, 2015). Clinically, mTBI is defined as a mild insult to the head causing a brief (<30 min) period of unconsciousness and/or confusion and disorientation (Arciniegas et al., 2005; McInnes et al., 2017). Because of the absence of physical signs of severe injury, most mTBI patients are discharged without follow-up (Mayer et al., 2017). However, ∼10–25% of the patients with mTBI suffer long-term consequences such as depression, attention or memory problems, and sleep or mood disorders, which still persist one year after the injury (Grandhi et al., 2017).

Computed tomography (CT) and MRI are the gold-standard techniques for clinically assessing tissue damage after TBI (Kim and Gean, 2011). CT is commonly available and used in the acute phase of TBI for initial assessment of the damage severity (Mutch et al., 2016). Currently, MRI methods such as T1- and T2-weighted, susceptibility-weighted imaging, and diffusion-weighted imaging are used in routine clinical practice in both acute and chronic phases of head injury (Amyot et al., 2015). These MRI methods are sensitive to macroscopic damage such as parenchymal bleeding, edema, and penetrating injuries after moderate and severe TBI (Kim and Gean, 2011; Kinnunen et al., 2011). CT and conventional MRI approaches fail to detect widespread microscopic injuries such as axonal injury, however, which is commonly observed after mTBI in humans (Shenton et al., 2012) and characterized histologically in animals (Johnson et al., 2013).

Diffusion tensor imaging (DTI; Basser et al., 1994; Mori and Zhang, 2006) is widely used in experimental and clinical settings to investigate TBI due to its sensitivity to changes in the tissue microstructure (Pierpaoli et al., 1996; Sidaros et al., 2008; Laitinen et al., 2015; Sierra et al., 2015; Hutchinson et al., 2018; Tae et al., 2018). Several experimental *in vivo* DTI studies have characterized microstructural changes mainly in the acute phase of mTBI (Budde et al., 2013; Long et al., 2015; Wright et al., 2016; Li et al., 2016). The interpretation of changes in DTI metrics as an indicator of changes in the cellular level, however, is ambiguous. Only a few studies have combined DTI and histology, but only qualitative histologic analysis was applied with no direct link to the DTI metrics (Zhuo et al., 2012; Hylin et al., 2013; Singh et al., 2016; Tu et al., 2017; Herrera et al., 2017).

The aim of this study was to identify the spatial distribution of tissue damage throughout the rat brain in the acute and subacute phases of mTBI using *in vivo* MRI. As screening methods, we used *in vivo* voxel-wise and deformation-based morphometry analyses for DTI and T2-weighted MRI data, respectively, to assess progressive microstructural alterations and local morphologic volumetric changes throughout the brain. To interpret DTI changes in the tissue microstructure, we applied a structure tensor (ST) analysis for myelin-stained sections and an automated cell counting method for Nissl-stained sections to examine the contribution of both myelinated axons and cellularity to the DTI parameters.

## Materials and Methods

### Animals

Adult male Sprague Dawley rats (*n* = 25, 10 weeks old, weight 300–450 g, Harlan Netherlands B.V.) were used in all the experiments. All the animals were housed individually in cages and maintained in a climate-controlled room (temperature 22 ± 1°C, air humidity 50−60%) with a 12/12 h light/dark cycle and an *ad libitum* diet. All the experimental procedures were approved by the Animal Ethics Committee of the Provincial Government of Southern Finland and conducted in accordance with the guidelines set by the European Union Directives 2010/63/EU.

### Animal model of mild TBI

We induced mTBI using the lateral fluid percussion (LFP) injury model, as described previously (Kharatishvili et al., 2006). Briefly, rats were anesthetized by intraperitoneal injection (6 ml/kg) of a mixture of sodium pentobarbital (58 mg/kg), chloral hydrate (60 mg/kg), magnesium sulfate (127.2 mg/kg), propylene glycol (42.8%), and absolute ethanol (11.6%). We then performed a craniotomy (5 mm in diameter) between bregma and λ on the left convexity (anterior edge 2.0 mm posterior to bregma; lateral edge adjacent to the left lateral ridge). LFP injury was induced (*n* = 13) by a transient fluid pulse impact (21–23 ms) against the exposed dura using a fluid percussion device (AmScien Instruments). The impact pressure was adjusted to induce a mild brain injury (0.88 ± 0.21 atm). After the impact, we removed the animals from the fluid percussion device, and assessed the temporary absence of spontaneous breathing by measuring the apnea time. Additionally, we assessed the right-time reflex latency, presence of hematoma on the impact site and seizure post-mTBI. Sham-operated control animals (*n* = 12) underwent the same surgical procedures without the impact. Mild TBI animals recover same as the sham-operated animals after the impact, with no apnea, hematoma, or post-injury seizure. We also did not observe differences in right-time reflex latency in mTBI animals when comparing to sham-operated animals. The mortality during our experiments was 0%.

### *In vivo* MRI

All rats were scanned *in vivo* on days 3 and 28 after the injury under 1.0–1.5% isoflurane (in 70% nitrogen/30% oxygen) anesthesia. We performed *in vivo* imaging using a horizontal 7 T Bruker PharmaScan MRI system with an actively decoupled quadrature volume transmitter coil and a quadrature receiver surface coil pair (Bruker Biospin). We monitored the breathing and temperature of the rats during the scans using a respiration pneumatic sensor and a rectal temperature probe, maintaining physiologic stability (breathing approximately at 60 bpm, temperature ∼37°C) with a water circulation system (ThermoFisher Scientific). *In vivo* DTI data were acquired using three-dimensional (3D) spin echo-planar imaging sequences with respiratory gating and the following parameters: repetition time = 1000 ms, echo time = 30 ms, number of averages = 1, field of view = 21.4 × 14.4 × 15.6 mm³, bandwidth = 300 kHz, matrix size = 142 × 96 × 52, b0 images = 4, 60 directions (δ = 4 ms, Δ = 11 ms, b-value = 2000 s/mm^2^) with a resolution = 0.15 × 0.15 × 0.30 mm³, and a scan time of 2–3 h. We acquired T2-weighted images using 2D fast spin-echo sequences with the following parameters: repetition time = 7800 ms, echo time = 40 ms, rapid acquisition relaxation enhancement factor = 8, number of averages = 8, field of view = 2.56 × 2.56 cm^2^, matrix size = 170 × 342, resolution = 75 × 150 μm^2^, number of slices = 52, slice thickness = 300 μm, and scan time = 22 min.

### Statistical analyses of *in vivo* MRI

In voxel-wise and deformation-based morphometry analyses, we used FSL randomize (Winkler et al., 2014) with 10,000 permutations and threshold-free cluster enhancement (TFCE; 3D-connectivity with standard parameters) to perform the statistical analyses. We used the permutation-based TFCE (Smith and Nichols, 2009) with the default parameters (H = 2, E = 0.5, C = 6), which performs family-wise error (FWE) multiple comparison correction to the cluster-enhanced statistic values. For further discussion how to interpret TFCE results, we refer to Smith and Nichols (2009) and Noble et al. (2020).

### *In vivo* MRI data analysis

For DTI analysis, we first converted the DTI data to Nifti format. Second, we preprocessed and performed all the analyses using tools in FMRIB Software Library (FSL 5.0.9; http://www.fmrib.ox.ac.uk/fsl/). Then, we performed simultaneous multiple correction for motion and eddy current distortions with the FSL eddy tool (Andersson and Sotiropoulos, 2016). For that, we used eddy unchanged parameters without the opposite phase encoding correction (topup). After eddy current corrections, we determined the diffusion tensors, and the respective eigenvalues (λ_1_, λ_2_, and λ_3_) of the diffusion tensors were calculated using FSL. We then generated fractional anisotropy (FA), axial diffusivity (AD), radial diffusivity (RD), and mean diffusivity (MD) maps (Pajevic and Pierpaoli, 1999). We also calculated linear (CL), planar (CP), and spherical (CS) anisotropy indices (Westin et al., 2002).

To determine the differences between the animal groups, we performed a voxel-wise statistical analysis of the whole brain between sham-operated and mTBI animals at both time-points. We created a study-specific FA template (the mean of five accurately registered sham-operated brain images). The template was then used in the affine and nonlinear [symmetric image normalization method (SyN); Avants et al., 2008] co-registration of sham-operated and mTBI brain images from both time-points using Advanced Normalization Tools (ANTs; http://stnava.github.io/ANTs/; Avants et al., 2011).

**Figure 1.**
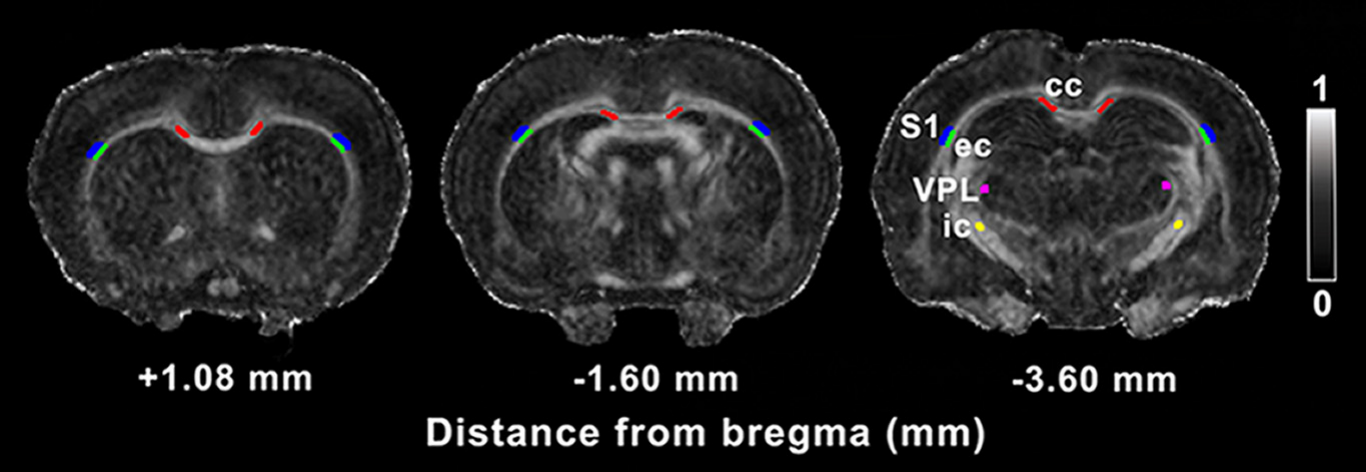
ROIs included in the DTI analysis. ROIs are outlined in a representative coronal FA map of a sham-operated animal. Gray scale indicates FA values between 0 (black) and 1 (white). cc, corpus callosum; ec, external capsule; ic, internal capsule; S1, somatosensory cortex; VPL, ventral posterolateral thalamic nucleus.

Additionally, based on the voxel-wise statistical analysis results on days 3 and 28 after injury, we used a region of interest (ROI) approach to correlate DTI data with the histologic analyses, selecting the same subgroup of animals used for the histologic procedures. In that subgroup, we manually outlined different brain areas at three different brain levels: two levels rostral to the lesion site, +1.08 and −1.60 mm from bregma, and one at the level of the lesion, −3.60 mm from bregma. We selected these three levels based on the absence or presence of major statistically significant differences in the DTI group analysis described above. The brain areas included in the ROI analysis were the ipsilateral and contralateral corpus callosum, external capsule, Layer VI of somatosensory cortex, internal capsule, and ventral posterolateral thalamic nucleus (Fig. 1). From each ROI, we extracted the FA and AD on day 28 post-mTBI as the only DTI parameters with statistically significant differences at this time point. The ROIs were outlined on FA maps from each individual animal using in-house MATLAB code (AEDES, http://aedes.uef.fi/; MATLAB R2012b).

T2-weighted data were analyzed using deformation-based morphometry to assess local morphologic volume differences throughout the brain (Gaser et al., 2001). We created a study-specific T2-weighted template image (the mean of six accurately registered sham-operated and mTBI brain images). Then, the individual images were co-registered to the template using ANTs linear and nonlinear SyN registration. To determine the changes in each voxel, we computed the Jacobian determinant by the inverse displacement vector field from the SyN registration using the CreateJacobianDeterminantImage script of ANTs. In this analysis, we compared sham-operated and mTBI rats on days 3 and 28 using FSL randomize.

At 3 d post-mTBI, we observed that the *in vivo* T2-weighted and DTI data showed blood-related susceptibility difference-artifacts related to the surgical procedure in seven sham-operated and mTBI animals, preventing satisfactory co-registration of some brain volumes for the T2-weighted deformation-based morphometry and DTI voxel-wise statistical analyses. From the 25 animals mentioned above (*n*_total_ = 25), we excluded six animals from the T2-weighted analysis (*n* = 19) and seven animals from the *in vivo* DTI analysis on day 3 post-mTBI (*n* = 18). For the same reason, on day 28 post-mTBI, we excluded three animals from the T2-weighted data analysis (*n* = 22) and two animals from the *in vivo* DTI analysis (*n* = 23).

### Tissue processing and histology

Thirty-five days post-mTBI, we anesthetized all rats with an intraperitoneal injection of urethane (1.25 g/kg, i.p., Sigma-Aldrich). Next, the animals were transcardially perfused with 0.9% NaCl for 5 min (30 ml/min), followed by 4% paraformaldehyde in 0.1 m PB, pH 7.4, for 25 min (30 ml/min). The brains were removed from the skull and postfixed in 4% PFA for 4 h. The brains were then placed in a cryoprotective solution (20% glycerol in 0.02 m potassium PBS, pH 7.4) for 36 h. After cryoprotection, we rapidly froze the brains in dry ice and stored them at −70°C until cutting. Using a sliding microtome, we sectioned a subgroup of brains (sham-operated = 6; mTBI = 8) in the coronal plane (30 μm, 1-in-5 series). We stored the first series of sections in 10% formalin, and the remaining series in cryoprotectant tissue-collecting solution (30% ethylene glycol, 25% glycerol in 0.05 m sodium phosphate buffer) at −20°C until histologic processing.

For the first series of sections, we performed Nissl staining (thionin) to assess the cytoarchitectonics, gliosis [increased cell density (CD)], and neurodegeneration after mTBI. In the second series of sections, we used gold chloride (Laitinen et al., 2010) to assess the myelinoarchitecture, myelin content, and axonal damage of the brain areas affected after mTBI. Briefly, sections mounted on gelatin-coated slides were incubated in 0.2% gold chloride solution (HAuCl_4_·3H_2_O, G-4022 MilliporeSigma) in 0.02 M sodium phosphate buffer (pH 7.4) containing 0.09% NaCl for 3–4 h at room temperature in the dark. We then washed the slides twice (4 min each) in 0.02 m sodium phosphate buffer containing 0.09% NaCl and incubated them in 2.5% sodium thiosulfate solution (5 min). After three washes in the buffer solution (10 min each), the slides were dehydrated (13 min), cleared in xylene (15 min), and cover-slipped with DePeX (Merck Millipore).

### ST analysis of myelin-stained sections

For the ST analysis, high-resolution photomicrographs of myelin-stained sections were acquired at a resolution of 0.013 μm^2^/pixel using a light microscope (Zeiss Axio Imager2) equipped with a digital camera (Zeiss Axiocam color 506). Each photomicrograph was analyzed using the ST-based method (Budde and Frank, 2012). To derive the anisotropy from the image, we used pixelwise ST analysis, where directional derivatives of an image were first produced by convolving the image with the directional derivative of a 2D Gaussian function (size = 11 pixels, σ = 3 pixels). Then, the ST for each image pixel was formed from the derivatives. These STs were summed into a pixelwise ST within a 128 × 128-pixel window, and the pixelwise tensor was eigen-decomposed. We used the eigenvalues to calculate the anisotropy index (AI). To compare the AI derived from ST-based analysis to the FA derived from DTI, we performed similar ROI analyses of the DTI data on myelin-stained sections, selecting the same brain areas at the locations mentioned above for the DTI data (Fig. 1). In the multiple linear regression analyses, we correlated MRI and histologic parameters obtained from white and gray matter areas individually and also using combinations of connected white and gray matter areas.

### CD analysis on Nissl-stained sections

We acquired high-resolution photomicrographs of the Nissl-stained sections and analyzed all the images using an in-house MATLAB code for automated cell counting analysis. Briefly, we developed an automated cell counting technique based on preliminary foreground segmentation and a subsequent segmentation error-correction strategy. For the preliminary cell segmentation, we applied Chan–Vese active contours (Chan and Vese, 2001) as implemented in MATLAB. The Chan–Vese model was initialized with a random binary mask. The speed function was the gradient of the green channel of the RGB images; the cell membranes appeared sharper in the green channel than in the blue and red channels. We set the smooth factor equal to 0.2, and the maximum number of iterations to 300 to perform the segmentation. The initial contours were deformed on the speed function to adapt to the cell shape, resulting in the preliminary segmentation of cells. The preliminary segmentation, however, contained under-segmented components (cells touching each other appear in the same connected component) and very small noise components. Thus, we fitted an ellipse to every preliminary component. If the major axis of an ellipse was smaller than a threshold [S = 30], the component was discarded, and if the major axis was greater than a threshold [B = 140], the component was recognized as an under-segmented component. The preliminary segmented components with the major axis of the fitted ellipse in [S, B] were correctly segmented, requiring no further analysis. We ran a secondary segmentation on every preliminary under-segmented component using the marker-based watershed transform. We determined the markers by first Gaussian filtering the intensity image obtained from the HSV transform of the RGB image. Within the domain of an under-segmented component, we defined the regional maxima of the H-maxima transform of the filtered image as markers. We applied a set of conditions to the extracted markers: (1) the value of a marker on the intensity image should be greater than a threshold [I = 0.5]; (2) the spatial distance between two markers should be greater than a threshold [D_s_ = 70]; and (3) the intensity distance between two markers should be greater than a threshold [D_i_ = 1]. Determining the set of markers, we applied a watershed transform to finalize the cell segmentation. We set the thresholds to experimentally evaluate the performance of the automated cell segmentation compared with 10 images in which all cells were counted by an expert (ISMM). We used the same threshold values for all images from different brain regions and experimental conditions. We defined the CD as ρ = N/A, where *N* is the number of cells in the ROI and A is the area of the ROI.

### Statistical analyses of the histology

ROIs statistical analyses of ST and CD histologic parameters were performed with GraphPad Prism (version 5.03, GraphPad Software Inc.). All values were expressed as mean ± SD. The unpaired *t* test was used to assess differences between both sham-operated and mTBI animals. Multiple linear regression was used to assess the relationship between DTI (FA and AD) and the histologic (AI and CD) parameters (regression model: DTI parameter ∼AI + CD). Multiple comparison correction was applied based on false discovery rate (FDR) using Benjamini–Hochberg procedure (Benjamini and Hochberg, 1995). To provide more information than just significant and non-significant division, we computed FDR-adjusted *p* values that we call *q* values according to the usual terminology. The FDR-adjustment was performed using the standard procedure introduced by Yekutieli and Benjamini (1999). We applied the FDR to correct for multiple comparisons separately in the unpaired *t* test and the multiple linear regression *p* values. The threshold for statistical significance was set to *q *<* *0.05.

## Results

Three days after the injury, eight of 13 mTBI rats showed hyperintensity indicative of edema at the injury site in the somatosensory cortex at −3.60 mm from bregma on T2-weighted images. Additionally, two of these eight rats also showed hypointensity in the cortex, indicating the presence of bleeding. In the subacute phase, six of 13 rats showed persistent cortical edema, but it was less pronounced compared with that at 3 d post-mTBI. Three of these six rats also showed bleeding.

### Whole-brain voxel-wise group analysis of *in vivo* DTI in the acute phase of mTBI

**Figure 2.**
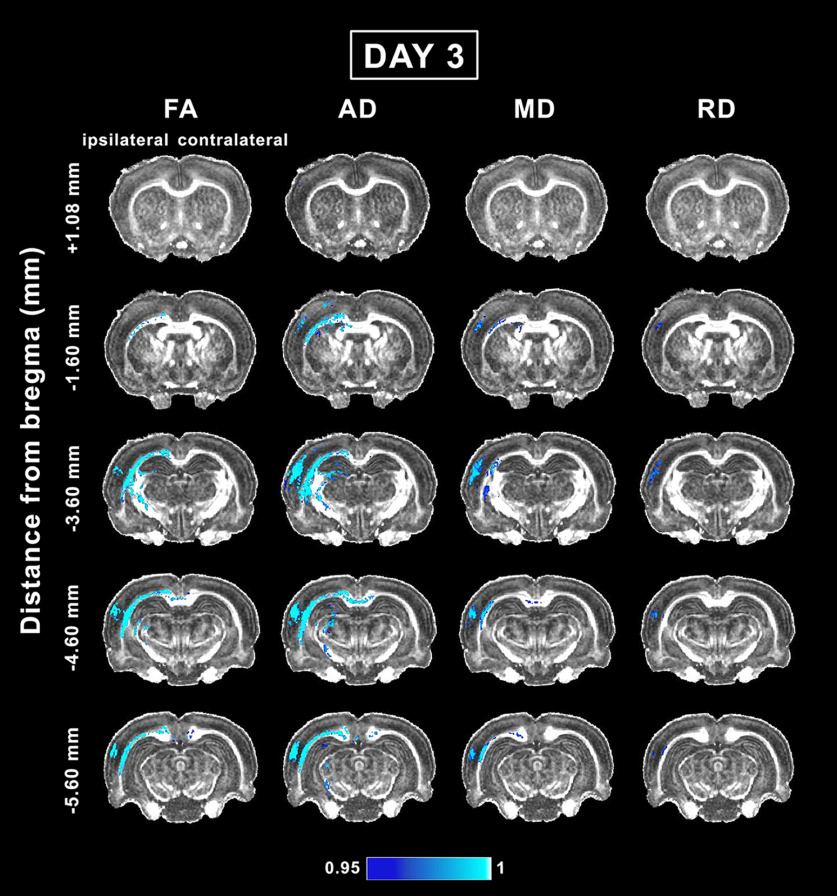
Whole-brain voxel-wise group analysis of FA, AD, MD, and RD parameters comparing sham-operated and mTBI animals at day 3. The mTBI rats showed significantly reduced FA, AD, MD, and RD parameters. The figure shows 1 – *p*, where *p* is the permutation-based FWE corrected *p* value after TFCE enhancement of the test statistic; a corrected *p *<* *0.05 was considered significant (blue-light blue color scale). AD, axial diffusivity; FA, fractional anisotropy; MD, mean diffusivity; RD, radial diffusivity.

**Figure 3.**
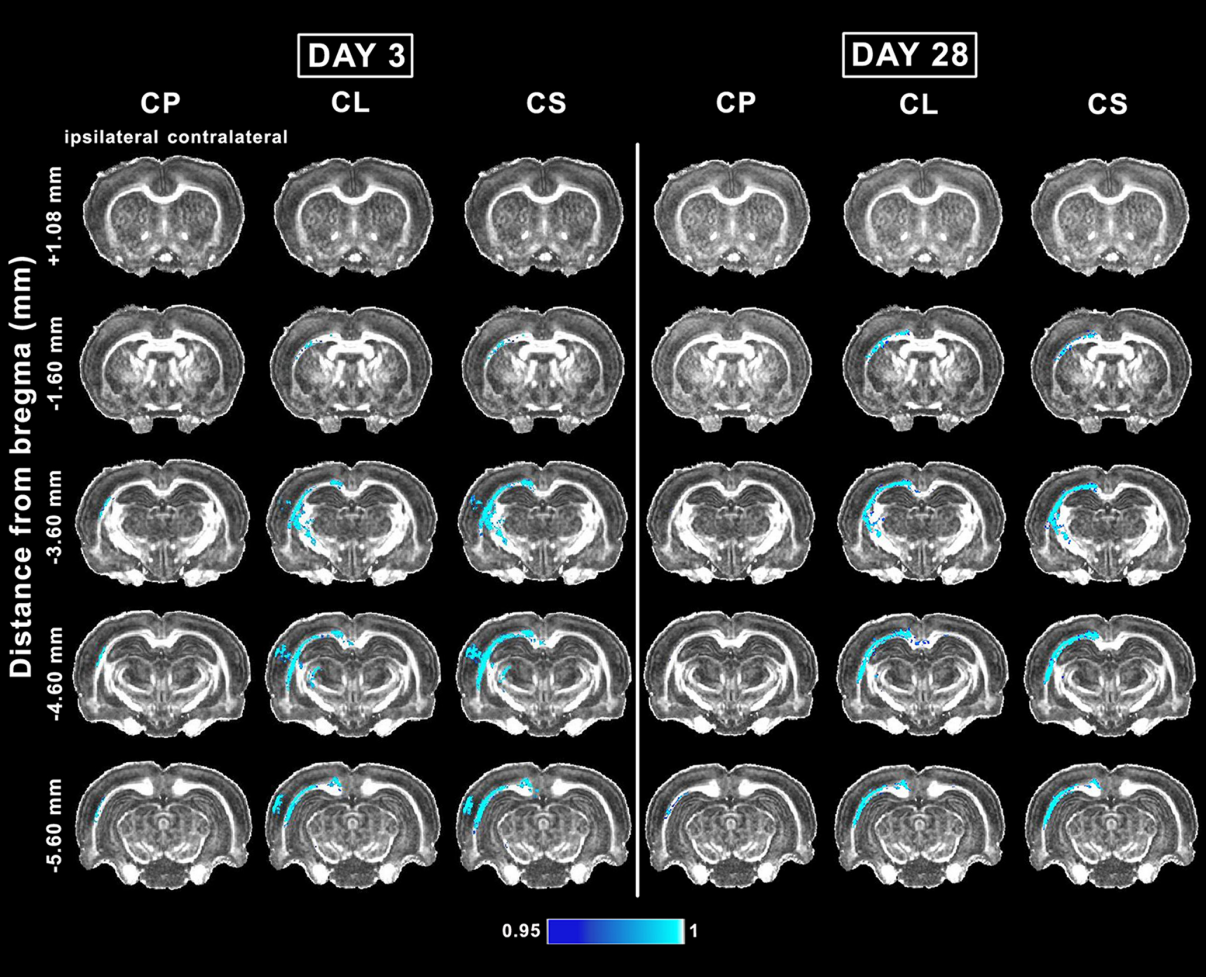
Whole-brain voxel-wise group analysis of Westin’s derived DTI parameters comparing sham-operated and mTBI animals at 3 and 28 d. The mTBI rats showed significantly reduced Westin’s derived DTI parameters. The figure shows 1 – *p*, where *p* is the permutation-based FWE corrected *p* value after TFCE enhancement of the test statistic; a corrected *p *<* *0.05 was considered significant (blue-light blue color scale). CL, linear anisotropy; CP, planar anisotropy; CS, spherical anisotropy indices.

At day 3 post-mTBI, voxel-wise group analysis revealed a decrease in the DTI parameters in both the gray and white matter, mainly ipsilateral to the injury. Rostrally, at −1.60 mm from bregma, we observed significant differences between the sham-operated and mTBI groups (Fig. 2). At this level, we observed significant decreases in FA, AD, and MD along the external capsule up to the cingulum. The AD and MD were significantly different between groups in the white matter, including the corpus callosum and fimbria, and in the gray matter in the somatosensory cortex, whereas differences in RD were only observed in the somatosensory cortex (Fig. 2). At the epicenter of the lesion, at −3.60 mm, FA, AD, and MD were decreased in white matter areas: the external capsule, corpus callosum, internal capsule, and cingulum. In gray matter areas, all the parameters were decreased in the somatosensory cortex, auditory cortex, and hippocampus (Fig. 2). Also, CL and CS significantly differed between groups in the corpus callosum, external capsule, internal capsule, and somatosensory cortex, whereas significant differences in CP were only observed in the external capsule (Fig. 3). At −4.60 mm from bregma, in addition to the structures mentioned at the previous level, the corpus callosum showed a significant ipsilateral decrease that extended to the contralateral side. Furthermore, the ventrobasal complex was highlighted in the FA, AD, CL, and CS analyses (Figs. 2, 3). More caudally at −5.60 mm, FA, AD, and MD were decreased in the external capsule, corpus callosum, and auditory cortex. AD was also decreased in the hippocampus and midbrain. Similarly to the previous level, RD was only decreased in the auditory cortex (Fig. 2). Furthermore, CL and CS exhibited significant differences in the corpus callosum, external capsule, and auditory cortex, whereas significant differences in the CP were only observed in the external capsule (Fig. 3).

### Whole-brain voxel-wise group analysis of *in vivo* DTI in the subacute phase of mTBI

**Figure 4.**
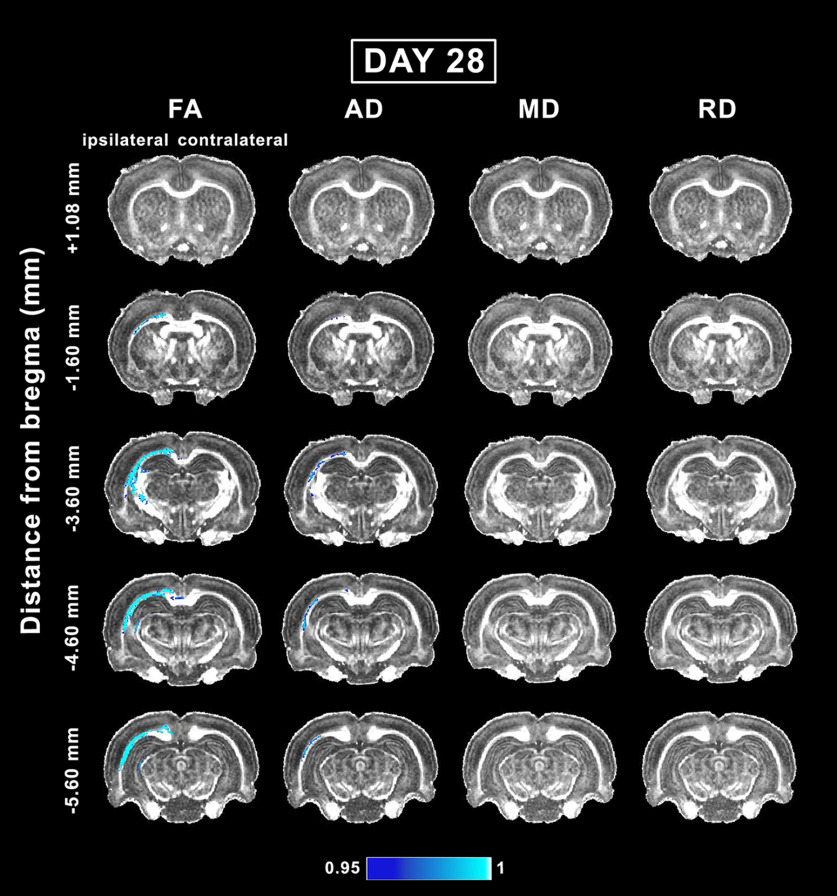
Whole-brain voxel-wise group analysis of FA, AD, MD, and RD parameters comparing sham-operated and mTBI animals at day 28. The mTBI rats showed significantly reduced FA, AD, MD, and RD parameters. The figure shows 1 – *p*, where *p* is the permutation-based FWE corrected *p* value after TFCE enhancement of the test statistic; a corrected *p *<* *0.05 was considered significant (blue-light blue color scale). AD, axial diffusivity; FA, fractional anisotropy; MD, mean diffusivity; RD, radial diffusivity.

At day 28 post-mTBI, voxel-wise group analysis revealed a decrease in DTI parameters mainly in the white matter ipsilateral to the injury. Overall, we observed no progression of the abnormalities between days 3 and 28. Rostrally in the brain, the analysis showed significant differences between groups at −1.60 mm from bregma (Fig. 4). At this level, the external capsule and cingulum exhibited a decrease in FA, AD, CL, and CS (Figs. 3, 4). At −3.60 mm, FA and AD were decreased in the corpus callosum, cingulum, and external capsule. FA was also decreased in the internal capsule and fimbria (Fig. 4). CL and CS were also significantly different between groups in the corpus callosum, external capsule, cingulum, and internal capsule (Fig. 3). Caudally at −4.60 mm, FA, AD, CL, and CS were significantly decreased in the external capsule and cingulum. In addition, FA and CL were decreased in the corpus callosum ipsilaterally and contralaterally (Figs. 3, 4). At −5.60 mm from bregma, FA, CL, and CS were decreased in the corpus callosum and external capsule. Additionally, AD and CP exhibited significant differences in the external capsule (Figs. 3, 4).

### T2-weighted deformation-based morphometry analysis after mTBI

To examine the intensity differences in T2-weighted images between sham and mTBI groups, we also performed intensity-based statistical analyses, and found no significant difference (*p* = 0.05, FWE-corrected TFCE-enhanced statistics).

**Figure 5.**
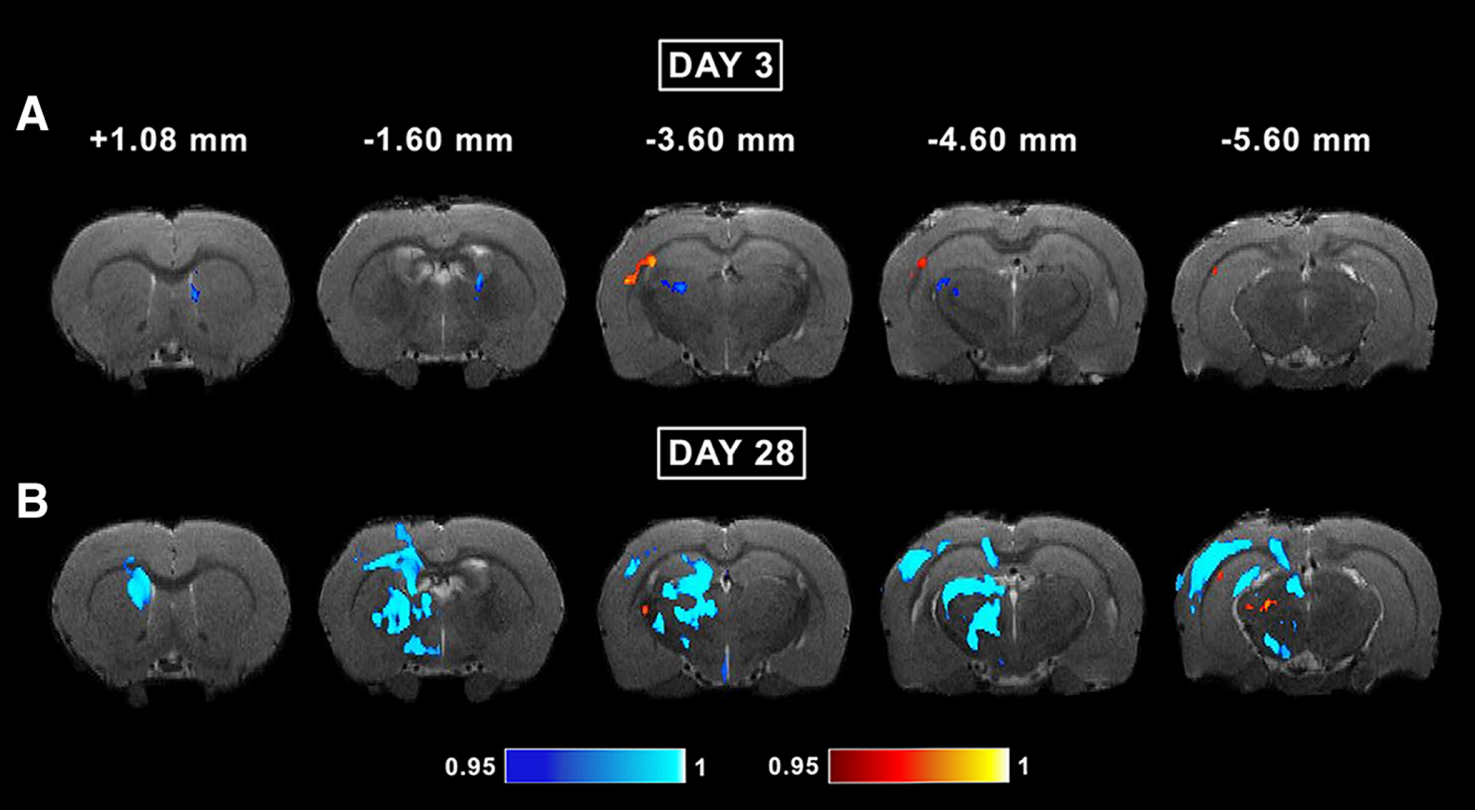
Whole-brain group deformation-based morphometry analysis of T2-weighted images comparing sham-operated and mTBI animals at day 3 (***A***) and 28 (***B***). Brain volume differences between sham-operated and mild TBI animals observed in acute and subacute phases post-mTBI. The mTBI rats showed both volume enlargement (red-yellow color scale), and volume reduction (blue-light blue color scale) compared with the sham-operated rats. The figure shows 1 – *p*, where *p* is the permutation-based FWE corrected *p* value after TFCE enhancement of the test statistic; a corrected *p *<* *0.05 was considered significant.

At day 3, mTBI animals showed decreased volume contralaterally in the lateral ventricle in the more rostral levels (Fig. 5*A*). At the epicenter of the lesion, the volume was increased in the ipsilateral external capsule and Layer VI of somatosensory cortex, and decreased in the ventrobasal complex after mTBI. At −4.60 mm, mTBI animals exhibited decreased volume ipsilaterally in the dorsolateral geniculate nucleus, and increased volume in the external capsule (Fig. 5*A*). Even at −5.60 mm from bregma, we still observed an increased volume in the external capsule.

At day 28 post-mTBI (Fig. 5*B*), the volume changes ipsilateral to the injury were more widespread compared with day 3 (Fig. 5*A*). In addition, the volume changes observed at day 3 were further progressed at day 28. Rostrally, mTBI animals exhibited decreased volume in the corpus callosum, cingulum, and caudate putamen. At −1.60 mm from bregma, the volume was decreased in the internal capsule, fimbria, and motor cortex (Fig. 5*B*). At −3.60 mm, mTBI animals showed decreased volume in the internal capsule, dentate gyrus, somatosensory cortex, and ventrobasal complex. At −4.60 mm, the volume was decreased in the cingulum, corpus callosum, ventrobasal complex, and somatosensory and auditory cortex (Fig. 5*B*). At −5.60 mm from bregma, mTBI animals exhibited decreased volume in the corpus callosum and dentate gyrus, and in the somatosensory, auditory and visual cortices. At this level, the medial geniculate nuclei showed an increased volume after mTBI.

### Qualitative and quantitative histologic assessment of myelin and Nissl-stained sections in the subacute phase of mTBI

For histologic assessment, we focused our analysis on areas highlighted in the group analyses, including the corpus callosum, external capsule, layer VI of somatosensory cortex, internal capsule, and ventral posterolateral thalamic nucleus both ipsilaterally and contralaterally. We analyzed these areas at two levels rostral to the lesion (+1.08 and −1.60 mm from bregma), and at the epicenter of the lesion (−3.60 mm from bregma) where the group analyses showed or did not show statistically significant differences.

The most devastating consequence after TBI is diffuse axonal injury caused by rapid acceleration/deceleration movements of the head (Marion et al., 2018). As a result, the axonal cytoarchitecture changes, progressing from a disruption in axonal transport to axonal swelling, secondary disconnection, and, finally, demyelination or Wallerian degeneration (Johnson et al., 2013). The alterations in myelinated axons at 35 d after injury may correspond with injured or degenerating axons, or demyelination.

Together with a qualitative examination of the histologic preparations, we performed quantitative assessments in both ipsilateral and contralateral hemispheres to compare sham-operated (*n* = 6) and mTBI animals (*n* = 8) by using ST analysis and automated cell counting on myelin-stained and Nissl-stained sections at day 35 post-mTBI. The results of the analysis are summarized in Table 1. We did not find any qualitative or quantitative differences when comparing histologic preparations from the contralateral side of sham-operated and mTBI animals (Table 1). Here, we focus our description on the comparison of the ipsilateral hemisphere between sham-operated and mTBI animals.

**Table 1:** Quantitative histologic analysis at +1.08, −1.60, and −3.60 mm from bregma

Level +1.08 mm									
*t* test		Ipsilateral				Contralateral			
		AI		CD (×10^−2^ cell/μm^2^)		AI		CD (×10^−2^ cell/μm^2^)	
		Mean ± SD	*t*(AI)	Mean ± SD	*t*(CD)	Mean ± SD	*t*(AI)	Mean ± SD	*t*(CD)
cc	Sham	0.81 ± 0.05	0.61	0.53 ± 0.03	0.41	0.80 ± 0.04	0.51	0.52 ± 0.04	0.38
	mTBI	0.83 ± 0.03		0.52 ± 0.04		0.81 ± 0.03		0.52 ± 0.02	
ec	Sham	0.73 ± 0.04	0.94	0.43 ± 0.03	0.62	0.75 ± 0.03	0.25	0.44 ± 0.05	0.47
	mTBI	0.71 ± 0.04		0.44 ± 0.04		0.75 ± 0.03		0.46 ± 0.05	
S1	Sham	0.43 ± 0.06	1.06	0.39 ± 0.02	0.56	0.47 ± 0.04	0.11	0.39 ± 0.01	0.80
	mTBI	0.47 ± 0.06		0.40 ± 0.06		0.47 ± 0.01		0.38 ± 0.03	
Level –1.60 mm									
cc	Sham	0.85 ± 0.02	2.06	0.51 ± 0.05	1.46	0.84 ± 0.04	0.04	0.50 ± 0.02	1.89
	mTBI	0.82 ± 0.04		0.55 ± 0.04		0.82 ± 0.03		0.53 ± 0.03	
ec	Sham	0.71 ± 0.06	1.91	0.43 ± 0.04	2.91	0.73 ± 0.02	0.51	0.42 ± 0.03	1.22
	mTBI	0.66 ± 0.03		0.50 ± 0.05		0.72 ± 0.05		0.44 ± 0.04	
S1	Sham	0.38 ± 0.05	0.23	0.39 ± 0.02	1.18	0.45 ± 0.05	0.52	0.39 ± 0.02	0.45
	mTBI	0.38 ± 0.06		0.41 ± 0.03		0.44 ± 0.05		0.39 ± 0.02	
Level –3.60 mm									
cc	Sham	0.83 ± 0.04	1.18	0.50 ± 0.06	0.50	0.80 ± 0.06	1.25	0.49 ± 0.03	0.37
	mTBI	0.78 ± 0.05		0.52 ± 0.06		0.75 ± 0.07		0.50 ± 0.06	
ec	Sham	0.76 ± 0.03	4.97	0.44 ± 0.03	4.71	0.73 ± 0.04	2.44	0.43 ± 0.03	0.47
	mTBI	0.60 ± 0.08*		0.56 ± 0.06*		0.78 ± 0.03		0.42 ± 0.03	
S1	Sham	0.34 ± 0.07	1.40	0.39 ± 0.02	4.01	0.35 ± 0.09	0.63	0.39 ± 0.03	1.04
	mTBI	0.30 ± 0.05		0.46 ± 0.05*		0.33 ± 0.04		0.38 ± 0.01	
ic	Sham	0.53 ± 0.09	1.40	0.38 ± 0.03	4.23	0.56 ± 0.08	0.23	0.38 ± 0.02	1.19
	mTBI	0.47 ± 0.04		0.45 ± 0.02*		0.57 ± 0.04		0.35 ± 0.04	
VPL	Sham	0.43 ± 0.03	0.49	0.33 ± 0.03	3.08	0.45 ± 0.05	0.28	0.33 ± 0.03	0.30
	mTBI	0.43 ± 0.03		0.38 ± 0.04		0.44 ± 0.04		0.34 ± 0.03	

Statistically significant FDR-corrected *q* values are shown (^*^ *q* < 0.05 corresponding to uncorrected ^*^ *p* < 3.10 × 10^−3^; unpaired *t* test) for the anisotropy index (AI) and CD (×10^−2^ cell/μm^2^). AI, anisotropy index; cc, corpus callosum; CD, cell density; ec, external capsule; ic, internal capsule; S1, somatosensory cortex; VPL, ventral posterolateral thalamic nucleus.

**Figure 6.**
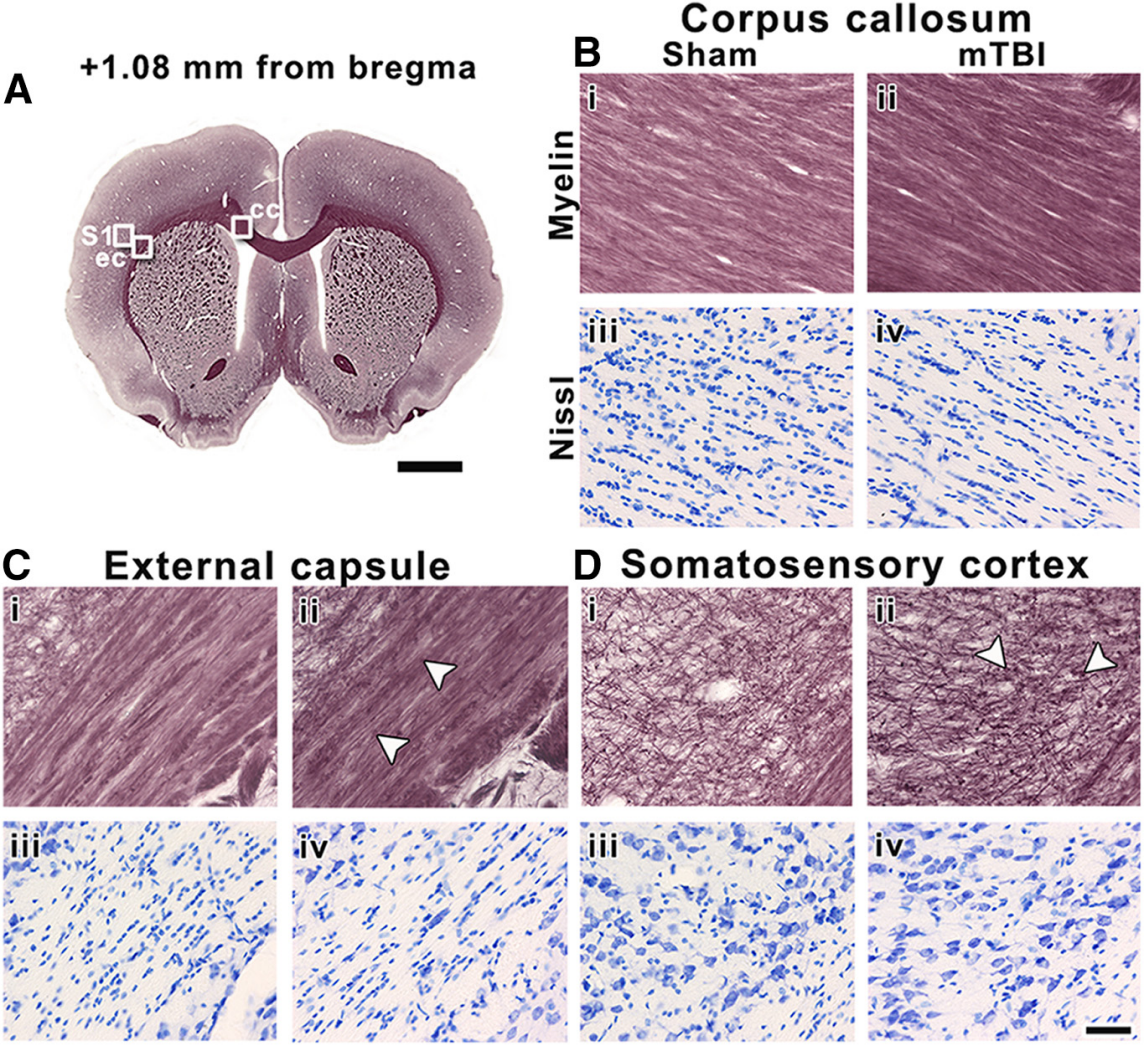
Representative whole-brain myelin-stained section of a sham-operated animal at +1.08 mm from bregma (***A***). White squares in ***A*** indicate the location of high-magnification photomicrographs of myelin-stained and Nissl-stained sections of a sham-operated (***i*** and ***iii***) and mTBI animal (***ii*** and ***iv***) in the corpus callosum (***B***), external capsule (***C***), and somatosensory cortex (***D***). The same animals are shown in both stainings. White arrowheads indicate axonal damage. cc, corpus callosum; ec, external capsule; S1, somatosensory cortex. Scale bars: 2 mm (***A***) and 50 μm (***B–D***).

Rostrally, at +1.08 mm, we observed alterations along myelinated axons in the ipsilateral external capsule and somatosensory cortex after mTBI (Fig. 6*Cii*,*Dii*), with no changes in cellularity (Fig. 6*Civ*,*Div*). Ipsilaterally in the corpus callosum, however, we observed no changes in the myelin or cellularity between mTBI and sham-operated animals (Fig. 6*Bi–iv*). We did not find significant differences between sham-operated and mTBI animals in any of these areas (Table 1).

**Figure 7.**
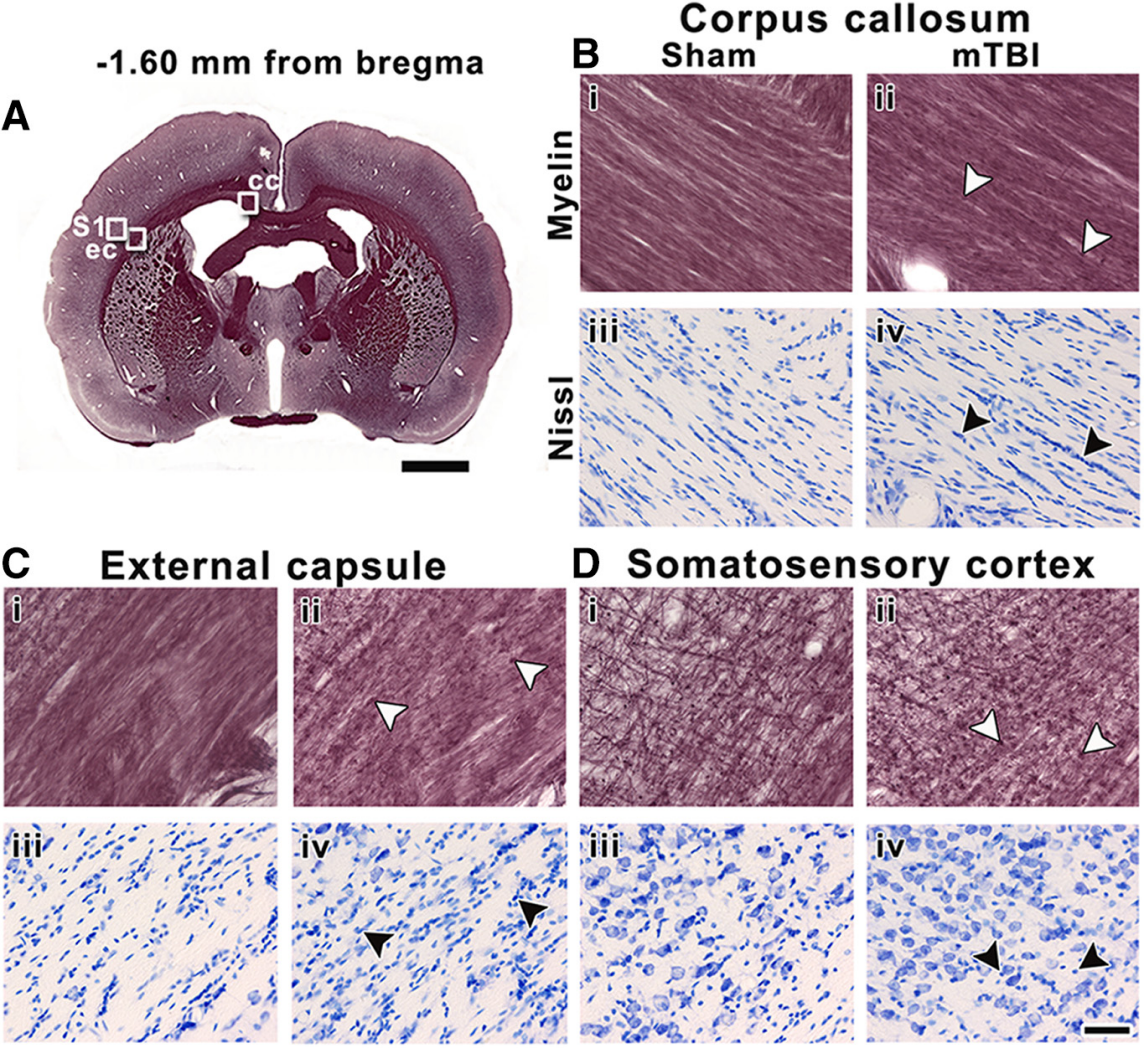
Representative whole-brain myelin-stained section of a sham-operated animal at −1.60 mm from bregma (***A***). White squares in panel ***A*** indicate the location of high-magnification photomicrographs of myelin-stained sections and Nissl-stained sections of a sham-operated (***i*** and ***iii***) and mTBI animal (***ii*** and ***iv***) in the corpus callosum (***B***), external capsule (***C***), and somatosensory cortex (***D***). The same animals are shown in both stainings. White arrowheads indicate axonal damage, and black arrowheads indicate gliosis shown by increased cellularity in Nissl-staining sections. cc, corpus callosum; ec, external capsule; S1, somatosensory cortex. Scale bars: 2 mm (***A***) and 50 μm (***B–D***).

At −1.60 mm from bregma, we observed more pronounced tissue alterations than at +1.08 mm. Here, mTBI animals showed mild axonal damage and gliosis in the corpus callosum (Fig. 7*Bii–iv*). We observed axonal damage and gliosis after mTBI in the external capsule and the somatosensory cortex (Fig. 7*C*,*Dii–iv*). Although some values showed a trend, especially the external capsule with lower AI and higher CD values comparing animal groups, we did not find significant differences between sham-operated and mTBI animals in any of these areas (Table 1).

**Figure 8.**
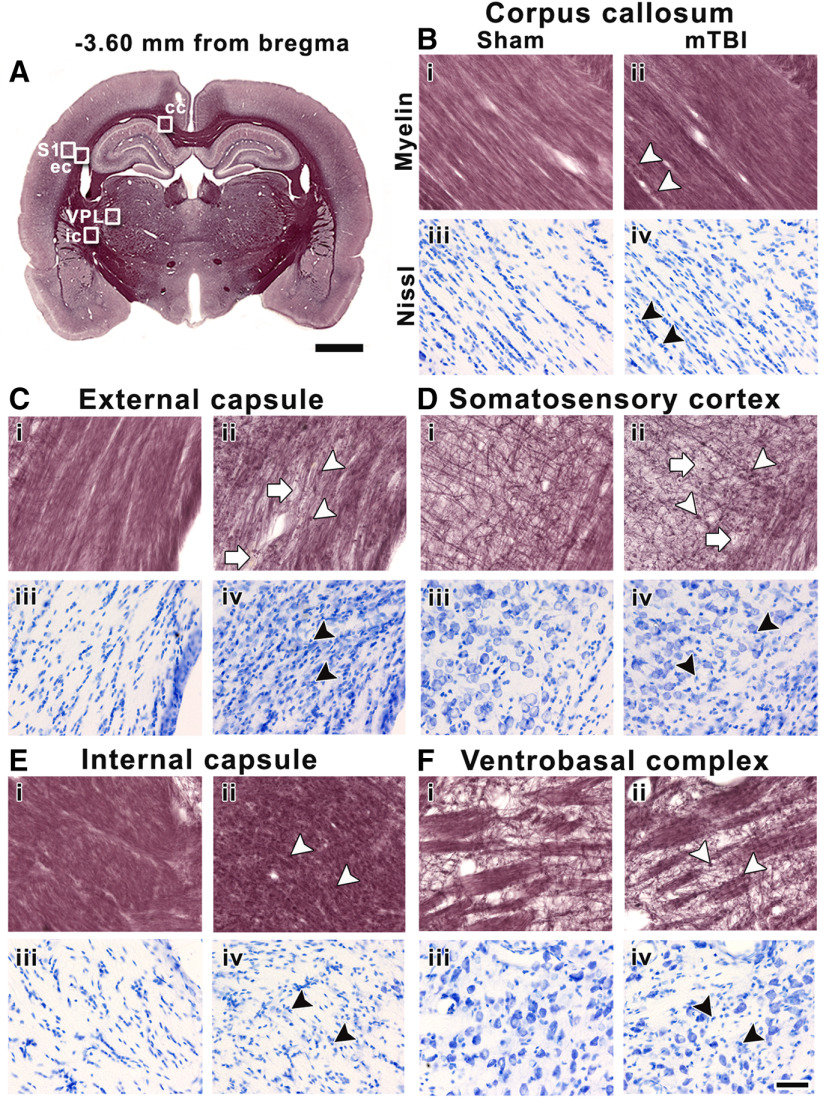
Representative whole-brain myelin-stained section of a sham-operated animal at −3.60 mm from bregma (***A***). White squares in panel ***A*** indicate the location of high-magnification photomicrographs of myelin-stained sections and Nissl-stained sections of a sham-operated (***i*** and ***iii***) and mTBI animal (***ii*** and ***iv***) in the corpus callosum (***B***), external capsule (***C***), and somatosensory cortex (***D***), internal capsule (***E***), and ventrobasal complex (***F***). The same animals are shown in both stainings. White arrowheads indicate axonal damage, and white arrows point to loss of myelinated axons. Black arrowheads indicate gliosis shown by increased cellularity in Nissl-staining sections. cc, corpus callosum; ec, external capsule; ic, internal capsule; S1, somatosensory cortex; VPL, ventral posterolateral thalamic nucleus. Scale bars: 2 mm (***A***) and 50 μm (***B–F***).

The analyzed areas showed more extensive alterations at the epicenter of the lesion than rostrally. In the corpus callosum, we observed widespread axonal damage (Fig. 8*Bii*) and increased cellularity (Fig. 8*Biv*) after mTBI; however, no significant differences were found in AI or CD when comparing sham-operated and mTBI animals (Table 1). In addition to the pronounced and widespread axonal damage, we observed a loss of myelinated axons and increased cellularity in the ipsilateral external capsule and somatosensory cortex in mTBI animals (Fig. 8*C*,*Dii–iv*) as compared with sham-operated animals (Fig. 8*C*,*Di–iii*). In the external capsule, we found a significant decrease in AI (*t* = 4.97, *q* = 1.76 × 10^−2^) which corroborated the damage and loss of myelinated axons, and a significant increase in CD (*t* = 4.71, *q* = 1.76 × 10^−2^) along with gliosis when comparing mTBI and sham-operated animals (Table 1). In the somatosensory cortex, we only found a significant increase in CD (*t* = 4.01, *q* = 3.41 × 10^−2^) corresponding with the presence of gliosis after mTBI (Table 1). Furthermore, in the ipsilateral internal capsule and ventral posterolateral thalamic nucleus, we observed axonal damage (Fig. 8*Eii*,*Fii*) and increased cellularity (Fig. 8*Eiv*,*Fiv*) after mTBI. We found a significant increase in CD in the internal capsule, indicative of gliosis in mTBI animals when comparing to sham-operated (*t* = 4.23, *q* = 3.41 × 10^−2^; Table 1).

### Multiple linear regression of DTI and histologic parameters

**Figure 9.**
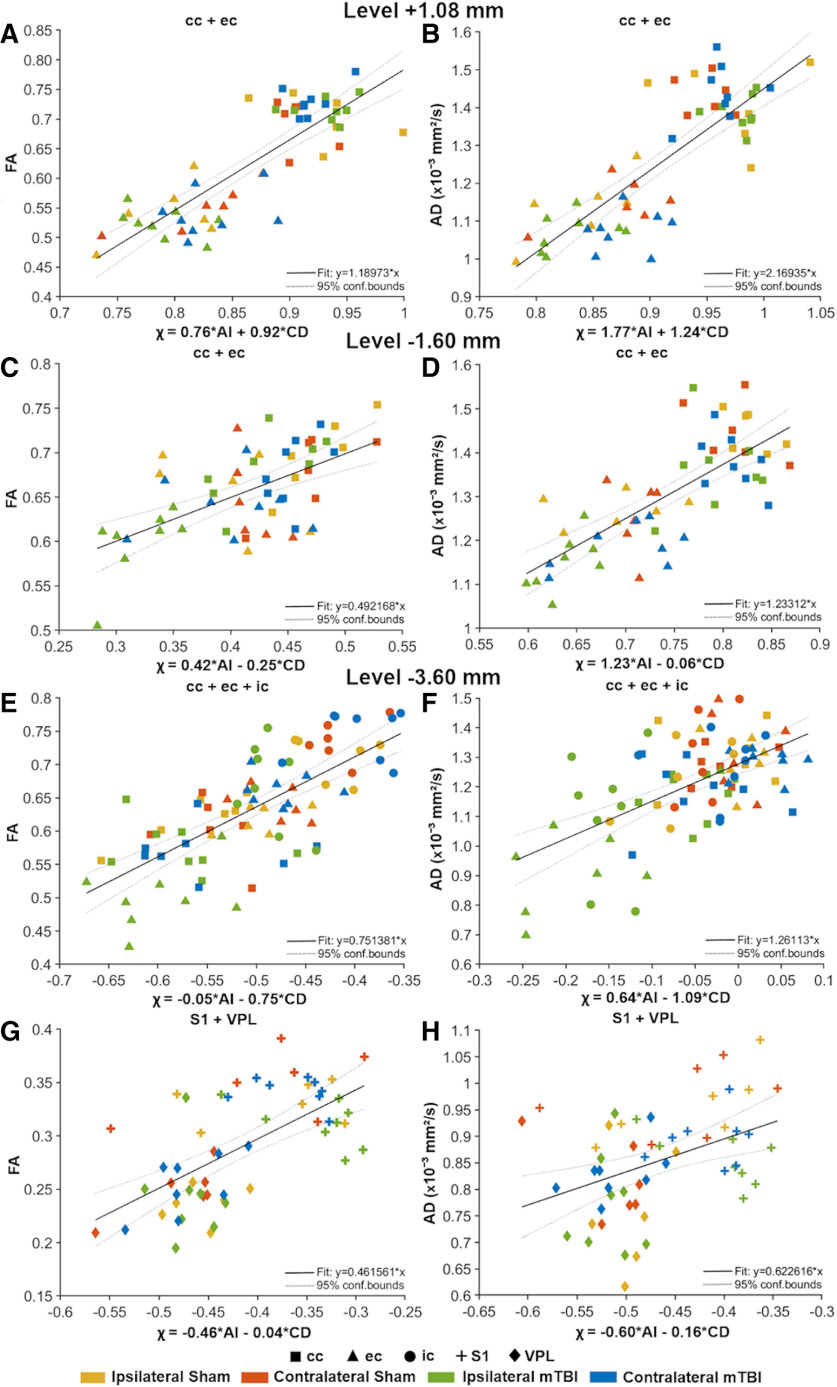
Representative multiple linear regression analyses of quantitative DTI and histologic analysis parameters at +1.08 mm (***A***, ***B***), −1.60 mm (***C***, ***D***), and −3.60 mm (***E–H***) from bregma. The thick black line is the regression line, and the two thin dotted lines represent the 95% confidence interval. Both sham-operated and mTBI animals (ipsilateral and contralateral hemispheres) are represented by colors, and the brain areas by shapes. The *x*-axis represents the χ values obtained with the expression: χ = β_AI_ × AI + β_CD_ × CD, where β is the weighting value. The *y*-axis represents FA or AD (×10^−3^ mm^2^/s). CD values are scaled (×10^−2^ cell/μm^2^). AD, axial diffusivity; AI, anisotropy index; cc, corpus callosum; CD, cell density; ec, external capsule; FA, fractional anisotropy; ic, internal capsule; S1, somatosensory cortex; VPL, ventral posterolateral thalamic nucleus.

We assessed the relationship between the DTI and histologic parameters from the ST and CD analyses in white and gray matter using a multiple linear regression test. Our regression model evaluated the contribution of two factors, AI and CD, to FA or AD (DTI parameter ∼AI + CD). Table 2 summarizes the outcomes of the multiple linear regression test, which includes the statistics of the model (*R* ^2^ adjusted and *F* statistic), the *t* statistics for AI and CD [*t*(AI) and *t*(CD)] and the corresponding *q* values. Figure 9 shows the most representative multiple linear regression results between FA or AD and χ = β_AI_ × AI + β_CD_ × CD, where β is the weighting value.

**Table 2:** Multiple linear regression between quantitative DTI and histologic analysis at +1.08, −1.60, and −3.60 mm from bregma

Level +1.08 mm								
	FA				AD (×10^–3^ mm^2^/s)			
	*R*² adj	*F* stat	*t*(AI)	*t*(CD)	*R*² adj	*F* stat	*t*(AI)	*t*(CD)
cc	0.02	1.24	–1.42	0.14	0	0.46	–0.20	–0.94
ec	0.11	2.64	1.38	1.88	0.15	3.45	2.58*	0.58
S1	0.01	1.09	–0.11	1.47	0	0.15	–0.16	–0.48
cc + ec	0.68***	60.0	5.10***	6.28***	0.66***	55.1	6.34***	4.53***
Level –1.60 mm								
cc	0.51***	14.8	1.49	–4.97***	0.09	2.25	–0.42	–2.12
ec	0.08	2.14	0.14	–1.94	0.25*	5.52	2.08	–1.87
S1	0	0.40	0.66	–0.55	0.05	1.65	–0.98	–1.60
cc + ec	0.33***	14.5	5.39***	–2.40	0.55***	34.3	7.54***	–0.28
Level –3.60 mm								
cc	0.20	4.29	2.91*	–0.78	0	0.87	1.17	–0.79
ec	0.72***	35.4	3.00*	–2.37	0.69***	31.4	4.54***	–0.32
S1	0.08	2.19	–0.35	–2.06	0.03	1.47	–0.36	0.10
ic	0.23*	4.95	1.98	–1.51	0.33**	7.76	3.72**	0.19
VPL	0.15	3.37	–1.96	–2.03	0	0.59	0.28	–0.98
cc + ec	0.53***	32.0	5.02***	–4.63***	0.48***	26.5	5.45***	–3.17**
cc + ec + ic	0.51***	44.2	–1.09	–8.52***	0.33***	21.6	5.44***	–5.23***
S1 + VPL	0.39***	18.6	–5.12***	–0.33	0.14*	5.48	–2.95*	–0.52

Statistically significant FDR-corrected *q* values are shown (*q < 0.05; **q < 0.01; ***q < 0.001 corresponding to uncorrected *p < 1.97 × 10^−2^, **p < 2.5 × 10^−3^, ***p < 1.22 × 10^−4^, respectively; multiple linear regression test) for the AI, CD (×10^−2^ cell/μm^2^), and both parameters. AD, axial diffusivity; AI, anisotropy index; cc, corpus callosum; CD, cell density; ec, external capsule; FA, fractional anisotropy; ic, internal capsule; S1, somatosensory cortex; VPL, ventral posterolateral thalamic nucleus.

Rostrally, at +1.08 mm, we found a correlation between FA and χ (*R* ^2^ = 0.68, *q* = 2.30 × 10^−12^; Fig. 9*A*) and AD and χ (*R* ^2^ = 0.66, *q* = 3.68 × 10^−12^; Fig. 9*B*) when combining the corpus callosum and external capsule (Table 2). In these two brain areas, FA (*t* = 5.10, *q* = 2.24 × 10^−5^) and AD (*t* = 6.34, *q* = 4.55 × 10^−7^) were positively associated with AI. We also found positive association between FA (*t* = 6.28, *q* = 5.09 × 10^−7^) and AD (*t* = 4.53, *q* = 1.36 × 10^−4^), and CD (Table 2). Despite the positive association between DTI and histologic parameters with AI and CD, we observed low influence of the injury in the correlations (Fig. 9*A*,*B*).

At −1.60 mm from bregma, FA correlated with χ in the corpus callosum (*R* ^2^ = 0.51, *q* = 2.13 × 10^−4^), while AD correlated with χ in the external capsule (*R* ^2^ = 0.25, *q* = 2.82 × 10^−2^; Table 2). When combining the corpus callosum and external capsule, FA correlated with χ (*R* ^2^ = 0.33, *q* = 4.10 × 10^−5^; Fig. 9*C*) and AD with χ (*R* ^2^ = 0.55, *q* = 5.39 × 10^−9^; Fig. 9*D*; Table 2). Also, we found that both FA (*t* = 5.39, *q* = 8.90 × 10^−6^) and AD (*t* = 7.54, *q* = 9.93 × 10^−9^) were positively associated with AI (Table 2). At this level, we observed a moderate influence of the injury in the relationship between DTI and histologic parameters, mainly in the external capsule (Fig. 9*C*,*D*).

At the epicenter of the lesion (−3.60 mm), we found a correlation between DTI and χ parameters in both white and gray matter areas (Table 2). In the external capsule, both FA (*R* ^2^ = 0.72, *q* = 4.55 × 10^−7^) and AD (*R* ^2^ = 0.69, *q* = 1.11 × 10^−6^) correlated with χ (Table 2). We found that both FA (*t* = 3.00, *q* = 1.80 × 10^−2^) and AD (*t* = 4.54, *q* = 4.35 × 10^−4^) in the external capsule were positively associated with AI (Table 2). Similarly, in the internal capsule, we also found a correlation between FA and χ (*R* ^2^ = 0.23, *q* = 4.13 × 10^−2^), and AD and χ (*R* ^2^ = 0.33, *q* = 7.91 × 10^−3^; Table 2). We found that only AD (*t* = 3.72, *q* = 3.51 × 10^−3^) was positively associated with AI (Table 2). When combining the corpus callosum and external capsule, FA (*R* ^2^ = 0.53, *q* = 1.04 × 10^−8^) and AD (*R* ^2^ = 0.48, *q* = 1.25 × 10^−7^) correlated with χ (Table 2). Also, we showed that both FA (AI: *t* = 5.02, *q* = 2.79 × 10^−5^; CD: *t* = −4.63, *q* = 9.88 × 10^−5^) and AD (AI: *t* = 5.45, *q* = 7.50 × 10^−6^; CD: *t* = −3.17, *q* = 8.09 × 10^−3^) were positively associated with both histologic parameters (Table 2). When combining the three white matter areas, we found a correlation between FA (*R* ^2^ = 0.51, *q* = 3.68 × 10^−12^; Fig. 9*E*) and AD (*R* ^2^ = 0.33, *q* = 3.15 × 10^−7^; Fig. 9*F*) with χ (Table 2). Here, FA (*t* = −8.52, *q* = 1.72 × 10^−11^) was negatively associated with CD (Table 2). Moreover, AD was positively associated with AI (*t* = 5.44, *q* = 3.78 × 10^−6^) and negatively associated with CD (*t* = −5.23, *q* = 7.49 × 10^−6^; Table 2). At this level, we found high influence of the injury in the correlation between DTI and histologic parameters as shown by the ipsilateral values of the three brain areas (Fig. 9*E*,*F*). In gray matter, we found that FA (*R* ^2^ = 0.39, *q* = 4.83 × 10^−6^; Fig. 9*G*) and AD (*R* ^2^ = 0.14, *q* = 1.99 × 10^−2^; Fig. 9*H*) correlated with χ when combining the somatosensory cortex and ventral posterolateral thalamic nucleus. Both FA (*t* = −5.12, *q* = 2.21 × 10^−5^) and AD (*t* = −2.95, *q* = 1.47 × 10^−2^) were negatively associated with AI (Table 2). Here, despite we found associations between DTI and histologic parameters, the influence due to the injury in these correlations was low (Fig. 9*G*,*H*).

## Discussion

In the present study, we investigated the potential of *in vivo* DTI to detect progressive widespread microstructural tissue alterations in white and gray matter areas throughout the brain after mTBI in rats. We performed quantitative histologic characterization of the *in vivo* DTI findings to unveil the underlying tissue processes associated with mTBI using ST and automated cell counting analyses in the subacute phase of mTBI. The key findings of this study were: (1) microstructural tissue alterations detected by voxel-wise analysis of DTI maps in white and gray matter areas progressed from day 3 to day 28; (2) volume reductions occur in white and gray matter areas in the subacute phase; (3) decreases in FA and AD are associated with axonal damage and gliosis in the subacute phase of mTBI; and (4) although we found a strong correlation between DTI and histologic parameters far from the lesion site, DTI was unable to detect mild axonal damage at this level. The combination of MRI and histologic analyses increases our knowledge of the potential of *in vivo* DTI to detect microstructural tissue changes in the brain after mTBI. This study may provide a new window for detecting progressive mild microstructural tissue damage using *in vivo* DTI.

### Microstructural alterations in the brain after mTBI

Three days after mTBI, our voxel-wise group analysis showed a decrease in FA, MD, AD, CL, and CS in major white matter areas, such as the corpus callosum, external capsule, and internal capsule in the ipsilateral hemisphere. Previous studies using *in vivo* DTI in a rat model of mTBI also reported changes in FA, AD, MD, and RD in white matter tracts in the acute phase (Long et al., 2015; Li et al., 2016; Wright et al., 2016; Herrera et al., 2017; Tu et al., 2017). Several studies report decreased FA in the corpus callosum at days 1 and 2 post-mTBI in the mild controlled cortical impact model (Long et al., 2015; Li et al., 2016; Tu et al., 2017) and increased RD (Long et al., 2015; Tu et al., 2017). Tu and collaborators also reported decreased AD and increased MD in the corpus callosum at day 1 post-mTBI (Tu et al., 2017). Further, using a mild impact acceleration model, Herrera and collaborators reported increased FA and MD in the fimbria and internal capsule and increased RD in the fimbria at day 3 post-mTBI (Herrera et al., 2017). Their study also showed decreased AD in the genu of the corpus callosum and fimbria, and decreased RD in the splenium of the corpus callosum. Using the mild LFP injury model, another study found decreased FA in the ipsilateral corpus callosum at 3 and 5 d post-mTBI, and increased RD at day 5 post-mTBI (Wright et al., 2016).

Consistent with our findings, a decrease in anisotropy and diffusivities in white matter were also observed in humans in the acute phase of mTBI (Arfanakis et al., 2002; Inglese et al., 2005). These studies reported decreased FA in the corpus callosum and internal capsule in the acute phase in mTBI patients. Inglese and collaborators also demonstrated increased MD in the internal capsule after mTBI (Inglese et al., 2005). In summary, findings from previous studies as well as the present study reveal that major white matter areas are extensively damaged in the acute phase of mTBI (Arfanakis et al., 2002; Inglese et al., 2005; Long et al., 2015; Li et al., 2016; Wright et al., 2016; Herrera et al., 2017; Tu et al., 2017). In particular, our study demonstrated consistent decreases in both anisotropy and diffusivities in the corpus callosum, external capsule, and internal capsule.

Our findings in gray matter areas revealed decreased FA, AD, MD, and RD mainly in the ipsilateral somatosensory and auditory cortices in the acute phase of mTBI. This is consistent with the work of Wright and collaborators (Wright et al., 2016) who also demonstrated decreased AD at 3 and 5 d post-mTBI in the ipsilateral cortex and increased trace at day 3 post-mTBI. While previous experimental and human studies revealed mainly white matter damage in the acute phase of mTBI, our findings revealed damage in both white and gray matter areas at an acute time point.

Twenty-eight days after mTBI, voxel-wise group analysis revealed microstructural alterations mainly in major white matter areas detected by *in vivo* DTI. We detected both decreased FA and AD ipsilaterally in the corpus callosum and external capsule and decreased FA in the internal capsule in the subacute phase post-mTBI. In these white matter areas, the decreases in CL and CS corroborated the decrease in FA at day 28 post-mTBI. Until now, only one study using a mild blast injury model in rats reported changes in the DTI metrics at day 30 post-mTBI using *in vivo* DTI (Budde et al., 2013). Laitinen et al. (2015), using a severe form of the LFP injury model, also demonstrated microstructural tissue changes in white matter areas, which included the genu of the corpus callosum, the angular bundle, and the internal capsule several months after injury. Human studies also showed progressive changes in the DTI metrics in white matter areas in the subacute phase in mild TBI patients (Rutgers et al., 2008; Messé et al., 2011). Messé et al. (2011) reported higher MD values in the corpus callosum in the subacute phase of mTBI between days 7 and 28 post-mTBI. In contrast, Rutgers et al. (2008) showed reduced FA predominantly in the corpus callosum and cingulum during the subacute and chronic phases of mTBI. In accordance with previous experimental and human studies, our results emphasize the persistent damage in white matter areas in the subacute phase of mTBI.

### Volumetric changes in the brain after mTBI

After TBI, neuronal damage induces anatomic as well as cellular morphology changes, leading to neurodegeneration (Harris et al., 2019). Neurodegeneration is associated with progressive volume loss after TBI detected by volumetric MRI measurements (Bigler, 2013; Cole et al., 2018). In our study, we assessed volume changes using T2-weighted deformation-based morphometry analysis. Our results revealed a volume reduction contralaterally in the lateral ventricle and ipsilaterally in the ventrobasal complex and increased volume in the external capsule and somatosensory cortex in the acute phase of mTBI. Previous studies revealed that a volume increase might be associated with edema early after mTBI (Mckee and Daneshvar, 2015; Stokum et al., 2016). In the subacute phase of mTBI, our results mainly showed a progressive volume reduction in the gray matter such as in the ipsilateral caudate putamen, hippocampus, ventrobasal complex, and cortical areas. In white matter areas, we also observed a volume reduction in the corpus callosum, cingulum, and internal capsule. After mTBI, a volume reduction in both white matter and gray matter areas is commonly observed in humans, and might indicate neural or axonal degeneration (Bigler, 2013; Mckee and Daneshvar, 2015). Until now, however, the few experimental studies that examined volumetric changes after mTBI using MRI volumetric analysis in rats revealed no brain volume changes (Kamnaksh et al., 2014; Wright et al., 2016). Kamnaksh et al. (2014), using DTI data in the mild blast injury model, reported no statistically significant volume changes in the hippocampus. On the contrary, we assessed local morphometric changes using T2-weighted images throughout the brain in the mild LFP injury model. Using the same animal model as in our study, Wright et al. (2016) did not find statistically significant total volume changes in the cortex, hippocampus, or corpus callosum using T2* data with ROI-based analysis; however, we reported local morphometric changes in the cortex and subcortical areas using T2-weighted deformation-based analysis.

In humans, the majority of studies that included MRI volumetric analysis performed after moderate and severe TBI reported volumetric abnormalities in the cortex, thalamus, putamen, and other regions (Gooijers et al., 2016; Cole et al., 2018). After mTBI, one human study also reported a volume reduction in cortical areas at one month after mTBI (Toth et al., 2013). Although Toth et al. (2013) observed a volume loss in cortical areas in the subacute phase of mTBI, they observed increased volume in the cortical gray matter and decreased volume in the ventricles and extracerebral cerebrospinal fluid at 3 d post-mTBI. We demonstrated a volume reduction in white and gray matter areas, which progressed from the acute to the subacute phase of mTBI, providing new insights in the detection of brain volume changes after mTBI.

### Histology reveals axonal damage and gliosis that correlate with DTI after mTBI

After the initial injury, an extensive complex cascade of molecular and cellular events occur, referred to as secondary injury (Jassam et al., 2017). Secondary cellular mechanisms include excitotoxicity and calcium flux, oxidative stress, mitochondrial dysfunction, and inflammation (Hill et al., 2016). This extensive secondary injury leads to cell death, impaired synaptic plasticity, and diffuse axonal injury, which compromise brain function (Walker and Tesco, 2013). Secondary damage after TBI has been assessed using DTI and histologic examination (Rodriguez-Paez et al., 2005; Mac Donald et al., 2007; Budde et al., 2011; Laitinen et al., 2015). To our knowledge, no other study has examined these changes using quantitative histologic analysis targeted on the basis of voxel-wise and morphologic MRI analyses. Previous studies demonstrated that decreased FA and AD parameters are widely associated with axonal injury after TBI (Mac Donald et al., 2007; Li et al., 2011; van de Looij et al., 2012). In the present study, we investigated the contribution of axonal damage and gliosis to the decreased FA and AD in the subacute phase of mTBI.

At 35 d post-mTBI, the most pronounced tissue changes were detected at the epicenter of the lesion, but we also observed alterations far from the primary lesion. Specifically, rostral to the lesion site, we detected axonal damage and increased cellularity (gliosis) in both white and gray matter areas such as the corpus callosum, external capsule, and somatosensory cortex. The DTI results correlated with the histologic parameters in the white matter, but not in the gray matter. At the lesion site, a previous study using a mild LFP injury model reported axonal injury in white and gray matter areas such as the corpus callosum, internal and external capsule, cingulum, and thalamus on day 15 after mTBI (Hylin et al., 2013). That study, using both astrocyte and microglia markers, also demonstrated inflammation in the corpus callosum and thalamus. Nevertheless, our study demonstrated the presence of axonal damage and gliosis in white and gray matter regions such as the corpus callosum, external capsule, internal capsule, somatosensory cortex, and ventral posterolateral thalamic nucleus. This reveals the plausible contribution of both tissue components to the decreases in FA and AD after mTBI. Notably, we found more extensive tissue changes in the external capsule and somatosensory cortex compared with the above-mentioned areas in association with the loss of myelinated axons. No alterations in the DTI metrics were found in the somatosensory cortex by *in vivo* DTI, which might be related to the complexity of the structure. Similar to the somatosensory cortex, *in vivo* DTI did not detect alterations in the ventral posterolateral thalamic nucleus, although the histologic findings in this area revealed axonal damage and gliosis. Nevertheless, our multiple linear regression test showed a correlation between DTI and histology in both the white and gray matter, suggesting that both axonal damage and cellularity may contribute to the DTI metrics. Furthermore, the correlations showed a greater influence of the injury toward the primary lesion in the brain, consistent with our group analysis results. These findings together demonstrate that axonal injury and gliosis contribute to the alterations in DTI metrics in both the white and gray matter after mTBI.

### Technical limitations and future directions

It is well known that the use of a simplistic tensor model in DTI underestimates the microstructure, especially in morphologically complex areas such as the gray matter (Tournier et al., 2011), which might explain the failure to detect mild microstructural changes in the somatosensory cortex and ventral posterolateral thalamic nucleus in this study. Therefore, future studies should focus on using more advanced diffusion MRI acquisitions and post-processing tools such as high-angular-resolution diffusion imaging, diffusion kurtosis, q-ball imaging, and neurite orientation dispersion and density imaging (Jensen et al., 2005; Kuo et al., 2008; Tournier et al., 2011; Zhang et al., 2012; Douglas et al., 2015). To quantitatively assess the DTI findings, we used ST analysis as a sensitive automated method offering anisotropy and orientation of histologic images equivalent to FA in DTI (Budde and Frank, 2012). In the present study, we used 2D histologic sections limiting the analysis to 2D, and therefore, missing the full 3D histopathologic interpretation. Future studies should focus on using ST analysis of 3D histologic images acquired by advanced 3D microscopy methods such as confocal, multi-photon, or electron microscopy (Schilling et al., 2016; Salo et al., 2018; Abdollahzadeh et al., 2019).

It is important to highlight that this study uses only male rats and it may limit the translation of our findings into the clinic (Rubin and Lipton, 2019). Therefore, future preclinical studies are needed to assess the effect of sex on mTBI outcomes.

## Conclusion

Our results indicate that *in vivo* DTI is sensitive for detecting widespread mild microstructural tissue alterations in white and gray matter areas throughout the brain in the acute phase of mTBI. In the subacute phase of mTBI, *in vivo* DTI detects progressive secondary tissue damage in the white and gray matter at the lesion site, but fails to detect changes distal to the primary lesion. We demonstrated that alterations in DTI metrics are associated with axonal damage and gliosis in both the white and gray matter. These findings offer new insights for imaging progressive tissue alterations after mTBI, highlighting the limitations of *in vivo* DTI for detecting mild microstructural damage, but opening up new ways to investigate tissue damage after mTBI.

## Synthesis

Reviewing Editor: Julie Bakker, University of Liege Decisions are customarily a result of the Reviewing Editor and the peer reviewers coming together and discussing their recommendations until a consensus is reached. When revisions are invited, a fact-based synthesis statement explaining their decision and outlining what is needed to prepare a revision will be listed below. The following reviewer(s) agreed to reveal their identity: NONE.

Both reviewers agree that the manuscript provides some advances in the field, but given the presentation, it is challenging to fully appreciate by how much. Specifically, given the description that a significant proportion of animals had edema (8/13), it is difficult to see if DTI is providing new information as a marker of mTBI, or if DTI is a reflection of the edema that is also seen with T2-weighted imaging.

Furthermore, the comprehensive and quantitative histological results are intriguing, but it is unclear from the current presentation how much novel insight the analysis/results provide. Specifically, additional structure tensor/cellularly maps to reveal the qualitative differences along with scatter plots of the resulting DTI/histological metrics would bring more clarity to the results as a comprehensive characterization of the relationships between the imaging and histological outcomes.

Comments from the reviewers:

Reviewer 1:

The manuscript reports a combined in-vivo DTI/T2-weighted morphometry/histology study of acute and subacute changes in white and grey matter throughout the brain, at the acute and subacute stages, following mTBI induced by the lateral fluid percussion injury model in adult male rats. In additional to presenting each imaging method separately, the degree of association between DTI and histological measures at day 28 was assessed (the novel aspect of this study) with mixed findings. Overall the manuscript is clear and well presented, although I have several moderate/minor concerns.

1. Introduction page 2 line 39-41 - “Many patients suffer long-term consequences, however, such as depression, attention or memory problems, and sleep or mood disorders that are usually not attributed to the injury (Grandhi et al., 2017).” This statement is poorly phrased and the meaning of “usually not attributed to the injury” is unclear.

2. Materials and methods page 4 line 72. Why were only male rats used in this experiment? The authors give no justification and there is no mention in the discussion. The use of male animals is a significant limitation that effects the translational utility of the findings.

3. Materials and methods page 4 line 87. How was the impact pressure validated as causing a mild brain injury (compared to moderate or severe)? Was the right-time reflex assessed? What was the mortality rate of the model?

4. Materials and methods page 5 line 109-111. - “We corrected the statistics maps for

multiple comparisons by threshold-free cluster enhancement (Smith and Nichols, 2009) with the following parameters (H = 2, E = 0.5, C = 6).” This statement is confusing as TFCE is used at the cluster forming level, correction for multiple comparison is achieved using FWE-corrected p values in FSL. Are the FTCE parameters different from the default settings? If so - why where they modified? Overall, the Statistical analysis of in vivo MRI section is difficult to follow. Results, such as those of the intensity difference in T2-weighted images, should be reported in the results section - even if there was no difference.

5. Materials and methods page 5 line 115-117. How was Eddy performed? Where phase encoding (PE) direction reversed B0 images also acquired? This information should be added to the in vivo MRI section

6. Materials and methods pages 6 line 124 - For the DTI template - which DTI-derived metric map was used? i.e. FA images?

7. Materials and methods pages 6 lines 124 and page 7 line 142 - For the template image, why were only control animals used in creating the DTI template, as this would bias the co-registration. Also, where the same animals used to create the T2-weighted template image? This is not specified in the text

8. Materials and methods pages 7 line 164 - why were only a subgroup of animals used for the histological analysis?

9. Materials and methods pages 10 line 224-225 - considering the number of regions examined, using a p-value with no correction for multiple comparisons results in an inflation of the likelihood on a type 1 error. FEW or FDR correction should be considered.

10. Results page 14-15 - and Table 1. Many of the significant linear regressions between the DTI metrics and the histologic metrics are for combined regions of interest i.e. corpus callosum + external capsule, S1 + VPL, etc. However, how and why these combined ROI’s were selected is not clear (nor is it described in the methods)

11. Discussion page 19 lines 433-435. The authors raise an interesting point. Could they comment on why they were able to observed volumetric changes in the current study, whereas previous reports observed no brain volume changes?

Reviewer 2:

This is a study investigating the effects of mild TBI in the rat as detected with diffusion tensor imaging and quantitative histological followup. Overall, the study interrogates a known feature (DTI is sensitive to microstructural injury and mTBI) in the context of gaps in understanding (DTI is insensitive to any single microscopic feature and the relationship with DTI-histology is challenging). As written, the study is largely descriptive and confirms the two above points.

The major limitation in my view is that the study reporting is somewhat superficial which makes it difficult to draw any strong conclusions. Several suggestions are given here to enhance the interpretations.

First, the effect of edema and T2 should be considered in the analysis since 8/13 animals were reported to have evidence of T2weighted edema. It is particularly relevant whether DTI is providing unique detection of injury or whether the DTI changes are simply a result of the edema visible on simpler and clinically used methods. Did the presence of T2 lesions change the contrast and registration accuracy, and did that affect the subsequent deformation based morphometry?

Second, there is limited visual reporting of the quantitative histological analysis. Specifically, it would be helpful to include maps of the structure tensor/cellularity for the regions investigated. If those are too course in resolution and don’t provide additional insight, inclusion of text within the figure panels with the tensor and/or quantitative value reported on each of the sections would help interpretation. On its own, it is difficult to appreciate many of the histological differences between sham/mTBI since they are subtle and only the qualitative changes are shown as figures.

Third, it would be useful to include scatterplots of selected DTI/histology relationships for easier interpretation of the results.

See additional points in the statistical analysis section of the online form.

Minor points:

Abstract:

"mTBI... long-lasting debilitating symptoms” is a claim that should be tempered to reflect results/data shown in the literature such as “approximately ## patients experience long-lasting symptoms” or similar as drawn from references cited in (Grandhi et al., 2017).

The hypothesis stating that “in vivo DTI detects progressive microstructural tissue damage..” isn’t fundamental insight and has been shown across many animal models and human studies. It should reflect the unique experiments/results presented by this study design and analysis.

Method:

Line 124 “we created a specific DTI template” should be “study-specific"

"well-registered” is ambiguous, how were these initially registered to create a template? Manually, affine..? or simply 5 happened to be aligned without any need for registration?

Results:

The results should state in plain language the directionality of each of the relationships “increased FA/AD associated with increased/decreased AI/CD/cellularity”. Or negative/positive associations.

"Histologic results” should use the specific term measured AD/CD.

Table 1 could be reorganized with R2, F, t(AI), t(CD) into a single row to make it easier to read.

For anatomical labels (+1.08. mm bregma) it might be also helpful to relate these to the injury epicenter in the figure labels to make the figure more readily understandable.

Figure 5 and 6 are sufficiently redundant that both need not be included (as primary figures). It is unclear what fig 5 provides that fig 6 does not also reveal and it wasn’t sufficiently understood what the uncorrected meant since I couldn’t find it in the methods.
